# *Legionella anisa* or *Legionella bozemanii*? Traditional and molecular techniques as support in the environmental surveillance of a hospital water network

**DOI:** 10.1007/s10661-023-11078-z

**Published:** 2023-03-22

**Authors:** Osvalda De Giglio, Marilena D’Ambrosio, Valentina Spagnuolo, Giusy Diella, Fabrizio Fasano, Carla Maria Leone, Marco Lopuzzo, Valeria Trallo, Carla Calia, Marta Oliva, Carlo Pazzani, Lucilla Iacumin, Sofia Barigelli, Maya Petricciuolo, Ermanno Federici, Francesco Paolo Lisena, Anna Maria Minicucci, Maria Teresa Montagna

**Affiliations:** 1 Interdisciplinary Department of Medicine, Section of Hygiene, University of Bari Aldo Moro, Piazza G. Cesare 11, 70124 Bari, Italy; 2 Department of Biomedical Sciences and Human Oncology, Section of Hygiene, Medical School, University of Bari Aldo Moro, Piazza G. Cesare 11, 70124 Bari, Italy; 3 Present Address: Section Hygiene - AOU Policlinico of Bari, Piazza G. Cesare 11, 70124 Bari, Italy; 4 Department of Biology, University of Bari Aldo Moro, Via Orabona 4, 70126 Bari, Italy; 5 Department of Agricultural, Food, Environmental and Animal Science, University of Udine, Via Sondrio 2/a, 33100 Udine, Italy; 6 Department of Chemistry, Biology and Biotechnology, University of Perugia, Via del Giochetto, 06122 Perugia, Italy; 7 Health Management, A.O.U. Policlinico of Bari, Piazza G. Cesare 11, 70124 Bari, Italy; 8 Regional Reference Laboratory of Clinical and Environmental Surveillance of Legionellosis, Interdisciplinary Department of Medicine, Section of Hygiene, University of Bari Aldo Moro, Piazza G. Cesare 11, 70124 Bari, Italy

**Keywords:** *Legionella non pneumophila*, *mip*-gene sequencing, PFGE, RAPD-PCR, Rep-PCR, SAU-PCR

## Abstract

Understanding the actual distribution of different *Legionella* species in water networks would help prevent outbreaks. Culture investigations followed by serological agglutination tests, with poly/monovalent antisera, still represent the gold standard for isolation and identification of *Legionella* strains. However, also MALDI-TOF and *mip*-gene sequencing are currently used. This study was conducted to genetically correlate strains of *Legionella non pneumophila* (*L-np*) isolated during environmental surveillance comparing different molecular techniques. Overall, 346 water samples were collected from the water system of four pavilions located in a hospital of the Apulia Region of Italy. Strains isolated from the samples were then identified by serological tests, MALDI-TOF, and *mip*-gene sequencing. Overall, 24.9% of water samples were positive for *Legionella*, among which the majority were *Legionella pneumophila* (*Lpn*) 1 (52.3%), followed by *Lpn*2-15 (20.9%), *L-np* (17.4%), *Lpn*1 + *Lpn*2-15 (7.1%), and *L-np* + *Lpn*1 (2.3%). Initially, *L-np* strains were identified as *L. bozemanii* by monovalent antiserum, while MALDI-TOF and *mip*-gene sequencing assigned them to *L. anisa*. More cold water than hot water samples were contaminated by *L. anisa* (*p* < 0.001). PFGE, RAPD, Rep-PCR, and SAU-PCR were performed to correlate *L. anisa* strains. Eleven out of 14 strains identified in all four pavilions showed 100% of similarity upon PFGE analysis. RAPD, Rep-PCR, and SAU-PCR showed greater discriminative power than PFGE.

## Introduction

*Legionella* are Gram-negative bacteria that can be found in natural (Atkinson et al., 2022; Zhan et al., 2022) and artificial aquatic environments (Prussin et al., 2017; Volker et al., 2016). The genus includes 64 species and more than 70 different serogroups (LPSN, 2022). *Legionella* are responsible for legionellosis, an infectious disease acquired by inhalation or aspiration of aerosols from contaminated water (De Giglio et al., 2019). The presence of amoebas, biofilms, and stagnant water and a temperature between 37 and 42 °C promote the growth of this microorganism (Van der Koiji et al., 2016). However, it is able to adapt to a wide range of water parameters, including temperatures of 5.7–63 °C, pH values of 5.5–8.1, and oxygen concentrations of 0.3–8.2 mg/L (Fliermans, 1996; Schwake et al., 2021; De Giglio et al., 2021).

Recently, the new European Drinking Water Directive (Directive EU 2020/2184) has inserted *Legionella* as mandatory among microbiological parameters to detect in water intended for human consumption for the risk assessment of domestic distribution systems in health and community facilities.

In Europe, notification rates of Legionnaires’ disease vary from fewer than 0.5 cases to 5.7 cases per 100,000 of the population (ECDC, 2022). Although over 80% of human cases are caused by *L. pneumophila* (*Lpn*) serogroup 1 (*Lpn*1), the real number of *Lpn* non-serogroup 1 and *Legionella* non-*pneumophila* (*L-np*) cases (e.g., *L. micdadei*, *L. bozemanii*, *L. longbeachae*, *L. dumofii*, *L. feeleii*, and *L. anisa*) is poorly documented (Beauté et al., 2013; Craun et al., 2010; ESGLI, 2017; Girolamini et al., 2021; Head et al., 2019; Vaccaro et al., 2016; Yang et al., 2010). In particular, *L. anisa* is the most commonly detected *L-np* in aquatic environments, and rarely causes infections in humans (Doebelling et al., 1989; Akermi et al., 2006). *L. bozemanii* and *L. micdadei* which are not frequently found in the environment are the most common causes of culture-verified *L-np* infections (Doebelling et al., 1989; Svarrer & Uldum., 2012; Neiderud et al., 2013; Miller et al., 2007).

The risk of *Legionella* transmission with severe outcomes has been found to be affected by the complexity of hospital water systems and the vulnerability of hospitalized patients (De Giglio et al., 2021). Two thousand seven hundred and twenty-six cases of legionellosis were reported in Italy in 2021 with an incidence rate of 46 cases per 1 million of which 3.7% was of nosocomial origin (Istituto Superiore di Sanità, 2022). When it was possible to use culture-based methods, *Lpn* was identified in 100% of cases by Istituto Superiore di Sanità (2022). However, data describing the distribution of *L-np* in water systems of healthcare facilities are scarce (Arrigo et al., 2022; Girolamini et al., 2019, 2020, 2021; Mazzotta et al., 2021; Napoli et al., 2010). To our knowledge, there have been no reports of Italian legionellosis cases of nosocomial origin associated with *L-np*. However, it is likely that cases due to *L-np* are underestimated because the diagnostic methods commonly used in routine microbiological investigations are not sufficient for identification of species other than *Lpn*. Although culture-based method is considered the “gold standard” for microbiological surveillance of *Legionella*, it has some limitations in the identification of *L-np* (Scaturro et al., 2020). In recent years, many laboratories use a rapid identification method through the analysis of ribosomal protein pattern based on array-assisted laser desorption ionization mass spectrometry (MALDI-TOF MS) (Dilger et al., 2016; Pascale et al., 2020). Additionally, various genotyping techniques such as total genomic DNA analysis and specific (*mip* gene) and internal gene sequencing are available for identification of *Legionella* (Svarrer and Uldum 2012).

However, the use of different molecular techniques (e.g., pulsed-field gel electrophoresis (PFGE), RAPD, Rep-PCR, and SAU-PCR) is required to assess whether clinical patient isolates correspond to environmental isolates (Linee Guida per la prevenzione e il Controllo della Legionellosi, 2015; Haroon et al., 2010; Corich et al., 2005; Yamamoto et al., 2003).

In this study, *L-np* strains isolated during environmental surveillance conducted for 13 months in four pavilions of a large hospital in Apulia region (southern Italy) were identified using traditional methods (i.e., culture and latex agglutination) and spectrometry (MALDI-TOF MS) in conjunction with *mip*-gene sequencing. The aim of the study was to genetically correlate strains isolated from the water networks of the different hospital pavilions using traditional molecular methods as PFGE compared with alternative techniques (RAPD, Rep-PCR, and SAU-PCR) not usually applied to *L-np* strains.

## Materials and methods

Our Environmental and Food Hygiene Laboratory (ACCREDIA n.1683), which is recognized as a Regional Reference Center in Apulia (Southern Italy), has conducted clinical and environmental surveillance of legionellosis in nosocomial and community facilities since 2001.

In January 2020, systematic monitoring of the water network of a large Apulian public hospital was started as part of the implementation program of a Water Safety Plan. The surveillance plan covered 33 separate buildings and provided for the quarterly microbiological control of 50% of the water supply points uniformly distributed in each pavilion.

### Sampling and culture-based investigation

From 1 March 2021 to 30 March 2022, 346 water samples (1 L) were collected from taps of water system of four pavilions: pI (81), pII (109), pIII (128), and pIV (28). In detail, 277 samples were collected from the hot water systems and 69 from the cold water networks when it was not possible to obtain hot water samples (e.g., due to exhaustion of hot water in the boilers). The temperature was recorded for each water sample. According to Italian Guideline (Linee guida per la Prevenzione e il Controllo della Legionellosi, 2015), the collected samples were stored in sterile dark glass bottles containing sodium thiosulfate pentahydrate (0.01%, w/v) to neutralize chloride present in water. Samples were then transported, by an isothermal container, to the laboratory at room temperature (19.2 °C; range 18.5–24.2 °C).

According to ISO 11731:2017 (ISO 11731:2017), each sample was first filtered through isopore polycarbonate membranes (47 mm in diameter with a pore size of 0.22 µm) (Millipore Corporation, Bedford, MA, USA) then resuspended in 10 mL of the non-filtered water sample. After vortexing, an aliquot of each sample (200 µL) was seeded onto selective culture medium GVPC plates added with glycine, vancomycin, polymyxin, and cycloheximide B (Liofilchem Srl, Teramo, Italy) followed by incubation at 36 ± 2 °C for 7–10 days in a humid environment (to prevent desiccation of the plates) (ISO 11731:2017). Presumptive colonies (at least three different colonies for each plate) of *Legionella* were then inoculated in buffered charcoal yeast extract (BCYE) agar plates (BioMérieux, Marcy l'Etoile, France) with and without l-cysteine. The identification of the colonies of *Legionella* spp. grown only on BCYE agar plates added with cysteine was performed using a latex agglutination test with polyvalent (Biolife Italiana Srl, Milan, Italy) and monovalent antisera (Biogenetics Srl, Tokyo, Japan) (Linee guida per la Prevenzione e il Controllo della Legionellosi, 2015). In particular, regarding *Legionella* species group, the polyvalent antisera provided by the manufacturer (Biolife Italiana Srl, Milan, Italy) identify only few species implicated in clinical cases: *L. anisa*, *L. micdadei*, *L. bozemanii 1* and* 2*, *L. gormanii*, *L. longbeachae 1* and* 2*, *L. dumoffii*, and *L. jordanis*. Moreover, the monovalent sera commercially available allow to identify all *Legionella* species mentioned above except *L. anisa*, *L. longbeachae 1 and 2*, and *L. jordanis* (Biogenetics Srl, Tokyo, Japan).

All the strains identified as *Legionella* spp. were viewed under a long-wave ultraviolet light (360 ± 20 nm) emitted by a wood lamp to evaluate the emission of autofluorescence due to the presence of an intracellular pigment. The results of *Legionella* contamination in water samples were expressed as colony-forming units per liter (CFU/L), and the detection limit was 50 CFU/L.

### Identification by MALDI-TOF MS analysis and mip-gene sequencing

*Legionella* species strains were identified by MALDI-TOF using the Vitek MS system (BioMérieux, Italy) at level serogroup according to instruction of manufacturer. Also the *mip* gene sequence analysis was performed as reported by Federici et al. (2017). Briefly, thermal shock was used to extract genomic DNA from colonies of *Legionella* grown on BCYE medium, followed by PCR amplification of the *mip* gene. PCR reactions were set up on a total volume of 20 µL containing FIREPol 5 × Master Mix (Solis BioDyne, Tartu, Estonia), 0.4 µM of Legmip-f and Legmip-r primers (Ratcliff et al., 1998), and 4 µl of template DNA. PCR amplification protocol consisted of 34 cycles at 95 °C for 30 s of denaturation, 58 °C for 1 min of annealing, and 72 °C for 1 min of extension. At the end, amplicons were purified with the EuroSAP PCR Enzymatic Clean-up kit (Euroclone SpA, Pero, MI, IT) and sequenced by the Sanger method (Eurofins Genomics, Ebersberg; Germany). Sequencing results were analyzed and processed with Geneious Prime 2022.1.1 (Biomatters Inc., San Diego, CA, USA) running the BLAST (Basic Local Alignment Search Tool) tool to find similar *mip* genes belonging to *Legionella* reference strains in the GenBank database. A phylogenetic tree was constructed by the unweighted pair group method with arithmetic mean (UPGMA) method using Jukes-Cantor as a genetic distance model after aligning the obtained *mip* gene sequences with those of the culture collections strains downloaded from GenBank. Sequence alignment was performed using the multiple sequence comparison by log-expectation (MUSCLE) program with default settings (Edgar, 2004).

### Genetic correlation of Legionella non-pneumophila strains

Only 14 (C, F, G1, G2, G3, G4, H, I, L, M, P, R, S, and T) strains isolated from the water networks of the four different pavilions (pI, pII, pIII, and pIV) were possible to analyze establishing a possible genetic correlation. Strains L, M, P, R, S, and T were isolated from pavilion pI; C from pavilion pII; H and I from pavilion pIII; and F, G1, G2, G3, and G4 from pavilion pIV.

### Pulsed-field gel electrophoresis

PFGE of the 14 strains was performed using a modification of the CDC PulseNet standardized PFGE protocol (Ribot et al., 2006; Sabrià et al., 2001).

Briefly, isolates were grown on BCYE agar plates at 37 °C for 48 h, after which they were suspended in cell suspension buffer (10 mM Tris, 1 M NaCl, pH 8.0). The cell suspensions were then adjusted to an optical density of 0.5–0.6 at a wavelength of 610 nm and mixed with equal volumes of 1.0% pulsed-field certified agarose (BioRad, Milan, Italy) and 10 µL of proteinase K (20 mg/mL). Next, the agarose plugs were transferred into cell lysis buffer (0.5 M EDTA, pH 8.0, 1% sarcosine, and 200 µg/ml of proteinase K) and incubated at 50 °C overnight. DNA embedded in the plugs was digested with 40 U of AscI or SfiI (New England Biolabs, Schwahlbach, Germany) at 37 °C for 4 h. Plugs of *Salmonella braenderup* strain H9812 were digested with 40 U XbaI (TaKaRa Bio, Dalian, China) and used in each gel as universal size standards. Fragments of DNA were separated in a CHEF DR III System (BioRad Laboratories, Richmond, CA, USA) with a constant voltage of 6 V cm^−1^, an included angle of 120°, and increasing pulse times (5.6–50 s) at 14 °C for 21 h. All PFGE profiles were digitalized and the phylogenetic relationship was assessed through the fingerprinting software GelJ (Heras et al., 2015). The phylogenetic relationship was represented through a phylogenetic tree obtained using the Dice coefficient with clustering by the unweighted pair-group method with arithmetic mean (UPGMA). A 2% tolerance in band position differences was applied **(**Martínez-Puchol et al., 2021).

### RAPD, Rep-PCR, and SAU-PCR analyses

DNA extraction for PCR-based genetic fingerprint of the strains was performed using the GenElute™ Bacterial Genomic DNA Kit (Sigma-Aldrich, Milan, Italy) following the manufacturer’s instruction. The initial step was modified as follows: isolates were collected from *Legionella* agar plates (Sigma-Aldrich, Milan, Italy) using a 10-µL sterile loop, then resuspended thoroughly in 180 μL of lysis solution and the protocol was regularly followed. DNA was then quantified and standardized at 50 ng/µL (Nanodrop One system, Thermo Scientific, Marietta, OH, USA).

PCRs were performed using the amplification condition using a C1000 Touch Thermal Cycler (BioRad, Milan, Italy). The following primers were used for the reactions: M13 (5′-GAG GGT GGC GGT TCT-30′) (Huey & Hall, 1989), (GTG) 5 (5′-GTGGTGGTGGTGGTG-3′) (Gevers et al., 2001), and SAG1 (5′-CCGCCGCGATCAG-3′) for RAPD, Rep-PCR, and SAU-PCR, respectively. Conditions for PCR reaction, gel electrophoresis of the amplicons, and cluster analysis were performed according to Iacumin et al. (2006).

The 14 strains of *L-np* species were subjected to fingerprint analysis at least three times to confirm the reproducibility of the obtained profiles.

### Statistical analysis

The data were presented as numbers or percentages for the categorical variables. To determine if the distribution of load (cfu /L) of *Legionella* spp. was normal, a Shapiro–Wilk normality test was performed. Chi-square or Fisher’s exact test was performed to compare two or more mutually exclusive proportions or percentages in groups. The Kruskal–Wallis rank sum test and the Wilcoxon rank sum test were used to determine if water temperature influenced the presence of *Legionella* spp. and *L-np* species. To accomplish this, the water samples were grouped into negative and positive for *L-np* and *L. pneumophila* in the hot and cold water networks. R version 3.6.3 (The R Project for Statistical Computing, Vienna, Austria) was used for analysis. A *p-value* < 0.05 was considered to indicate statistical significance.

## Results

### Identification by agglutination test, MALDI-TOF MS technique, and *mip*-gene sequencing

Overall, 86/346 (24.9%) water samples were positive for *Legionella* spp. Analysis of positive samples using the polyvalent antiserum revealed that 45/86 (52.3%) samples were positive solely for *Lpn*1 (median value = 1,200 cfu/L; range 50–83,000 cfu/L), 18/86 (20.9%) for *Lpn* 2–15 (median value = 1,150 cfu/L; range 50–22,500 cfu/L), 6/86 (7.1%) for *Lpn*1 + *Lpn* 2–15 (median value = 980 cfu/L; range 450–4,400 cfu/L), 15/86 (17.4%) solely for *L-np* (median value = 200 cfu/L; range 50–13,000 cfu/L), and 2/86 (2.3%) for *L-np* + *Lpn*1 (median value = 905 cfu/L; range 410–1,400 cfu/L).

**Fig. 1:**
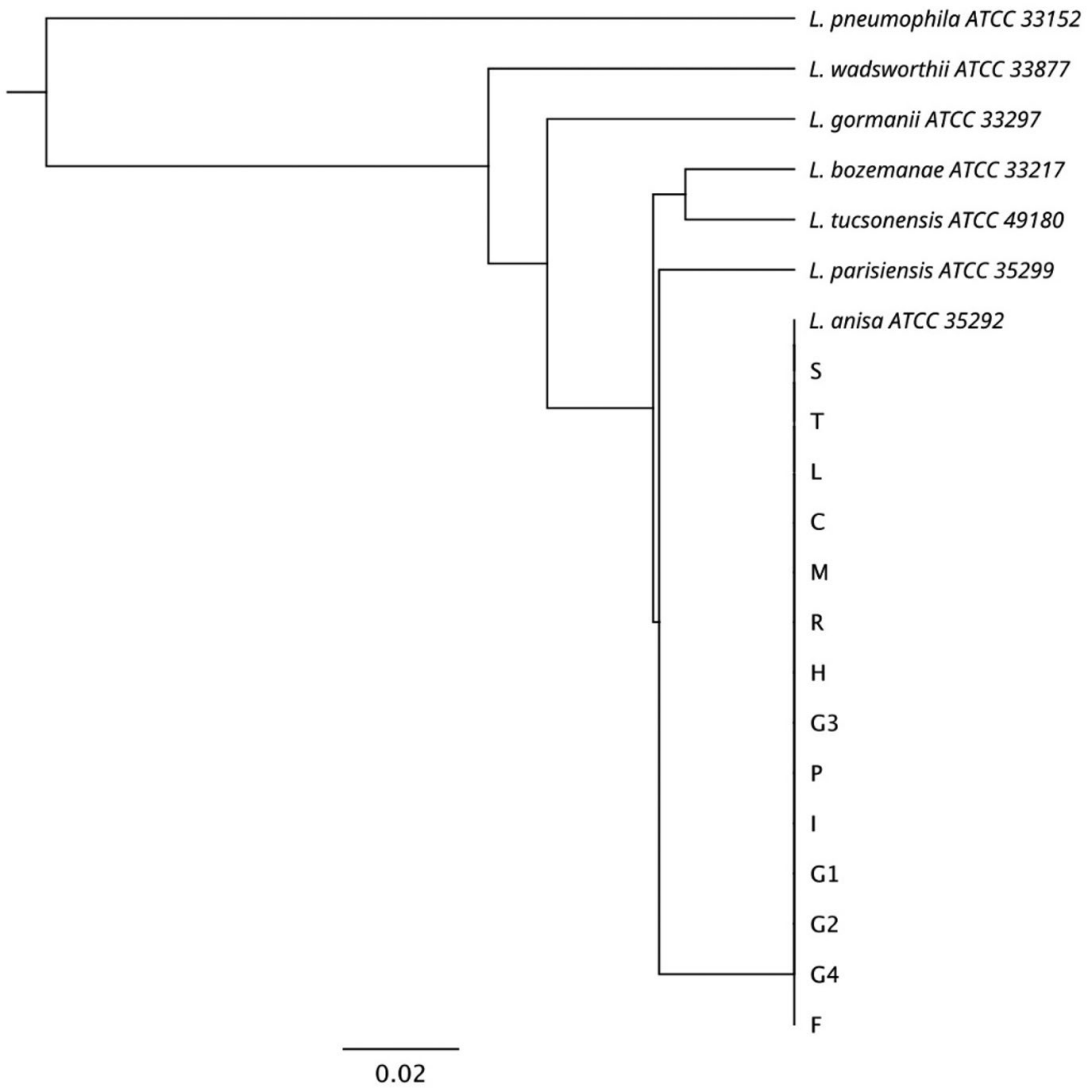
UPGMA phylogenetic tree showing similarities between the *mip* gene sequences of *Legionella* strains isolated in this study and those of the culture collections strains reported by the GenBank database

All colonies of *L-np* showed blue-white autofluorescence under UV light at 365 nm and were identified by monovalent antiserum as *Legionella bozemanii*. Subsequent MALDI-TOF analysis and *mip*-gene sequencing indicated that they were all *L. anisa* (Fig. 1). To assess the relationship between *L. anisa* isolates and the closest *Legionella* spp., a UPGMA-phylogenetic tree was built (Fig. 1) (Pascale et al., 2021). All isolates grouped into a single clade with the *L. anisa* ATCC strain, indicating 100% homology both among isolates and with the reference sequence. However, the remaining reference sequences were located in other clades, confirming the phylogenetic distance between *L. anisa* isolates and *L. bozemanii* (95.19% sequence identity).

Table 1 summarizes the distribution of isolates in the four pavilions. In particular, it shows two water samples revealed the presence of *L. anisa* mixed with *Lpn*1 in pII (*Lpn*1 200 cfu/L + *L. anisa* 210 cfu/L) and pIV (*Lpn*1 200 cfu/L + *L. anisa* 1,200 cfu/L).

**Table 1:** Distribution of hot/cold water samples positive for *Legionella* by species and pavilion (March 2021/March 2022)

**Pavilion** TP/TE (%)	***Lpn* 1** P/TP (%)	***Lpn* 2–15** P/TP (%)	***L. anisa*** P/TP (%)	***L. anisa* + *Lpn* 1** P/TP **(%)**	***Lpn* 1 + *Lpn* 2–15** P/TP (%)
**pI** 16/81 (19.8%)	4/16 (25%)	1/16 (6.3%)	9/16 (56.3%)	0/16 (0%)	2/16 (12.5%)
**pII** 49/109 (45%)	35/49 (71.4%)	10/49 (20.4%)	0/49 (0%)	1/49 (2%)	3/49 (6.1%)
**pIII** 8/128 (6.3%)	1/8 (12.5%)	4/8 (50%)	2/8 (25%)	0/8 (0%)	1/8 (12.5%)
**pIV** 13/28 (46.4%)	5/13 (38.5%)	3/13 (23.1)	4/13 (30.8%)	1/13 (7.7%)	0/13 (0%)
**Total** 86/346 (24.9%)	45/86 (52.3%)	18/86 (20.9%)	15/86 (17.4%)	2/86 (2.3%)	6/86 (7.0%)

*P* number of positive samples, *TE* number of examined samples, *TP* total positive samples

### Temperature analysis

The cold water network was found to have more *Legionella* contamination than the hot water network (39.1% vs 21.3%; *χ*^2^ = 9.40, *p-value* = 0.002). The same result was observed for *L. anisa* (44.5% cold water vs 5.1% hot water; Fisher’s exact test, *p-value* < 0.001). Conversely, more *Lpn* were present in the hot water system than the cold water system (94.9% vs 48.1%, *χ*^2^ = 22.68, *p*-value < 0.0001). No significant differences in the distribution of *Lpn* + *L. anisa* were observed between the hot and cold water systems (Fisher’s exact test, *p-value* = 0.09) (Table 2).

**Table 2:** Distribution of *Legionella* in hot and cold-water samples collected from water networks in the four investigated pavilions (March 2021–March 2022)

**Water** **network**	***Lpn*** **P/TP (%)**	***L. anisa*** **P/TP (%)**	***Lpn* + *L. anisa*** **P/TP (%)**	***Legionella*** **P/TP (%)**
**Hot**	56/59 (94.9)	3/59 (5.1)	0/59 (0.0)	59/277 (21.3)
**Cold**	13/69 (48.1)	12/69 (44.5)	2/69 (7.4)	27/69 (39.1)
**Total**	69/86 (80.2)	15/86 (17.4)	2/86 (2.3)	86/346 (24.9)

*P* number of positive samples, *TP* total positive samples, *TE* number of examined samples

Table 2 Distribution of *Legionella* in hot and cold water samples collected from water networks in the four investigated pavilions (March 2021–March 2022).

As shown in Table 3, *Legionella* distribution differed significantly among temperature ranges. Specifically, *L. anisa* was more often isolated from hot water samples with lower median temperatures than from other samples (Kruskal-Wallis *χ*^2^=12.545, *p-value*=0.001), i.e., positive for *Lpn* (pairwise comparisons using Wilcoxon rank sum test, *p-value*=0.05) and negative samples (pairwise comparisons using Wilcoxon rank sum test, *p-value*=0.04).

**Table 3:** Median values for water samples with different temperature ranges from hot and cold water networks (March 2021–March 2022)

	**Median value of temperature (°C)** **(range)**		
**Water network**	**Negative** **samples**	**Positive for** ***L. anisa***	**Positive for** ***Lpn***
**Hot**	50.1 (40.0–76.2)	41.5 (41.4–45.6)	47.1 (40.7–55.9)
**Cold**	20.2 (11.5–38.0)	19.1 (11.3–35.7)	24.1 (9.8–36.4)

For the cold water network, the median temperature of positive samples for *L. anisa* was lower than that of positive samples for *Lpn* vs. negative samples; however, this difference was not statistically significant (Kruskal–Wallis *χ*^2^ = 5.1781, *p-value* = 0.07

### Correlation among Legionella species strains

#### PFGE typing

**Fig. 2:**
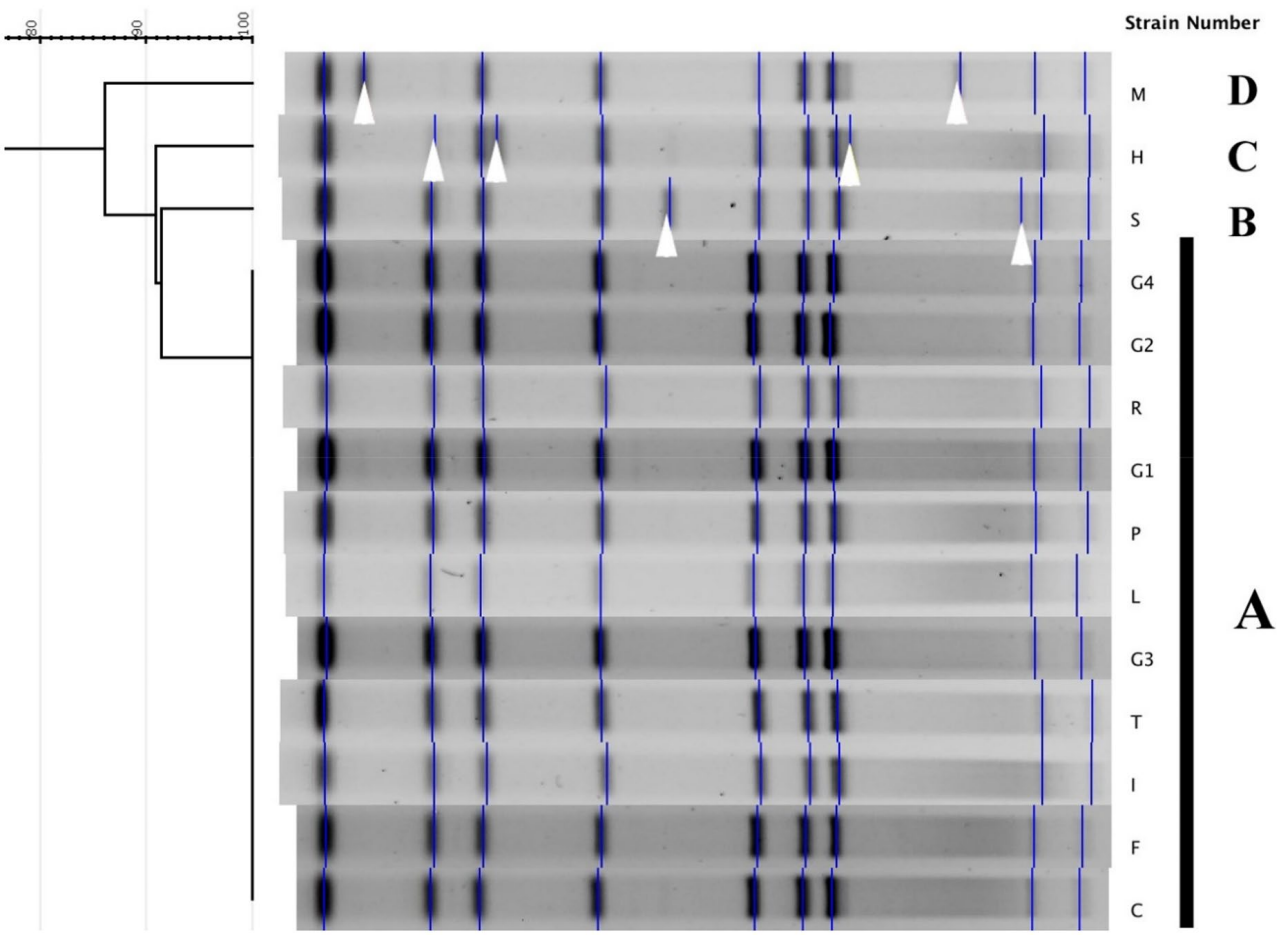
Dendrogram and PFGE patterns (generated by AscI) of 14 *Legionella anisa* strains. White arrows highlight different bands in profiles of groups B, C, and D compared with group A

Figure 2 shows the PFGE patterns after restriction with *AscI*. PFGE patterns differing by 1 or more bands were classified into single clusters. Eleven out of fourteen analyzed strains showed an indistinguishable PFGE profile and were clustered within the same group (termed A). Single strains assigned to groups B and C had > 90% similarity to group A and differed from the latter for 2 and 3 bands, respectively. Group D (also composed of a single strain) had 86% similarity to group A from which it differed for 3 bands. Analysis of PFGE profiles generated with *Sfi*I showed data comparable to that observed with *Asc*I. In particular, strains clustered in group A for *Asc*I showed an indistinguishable PFGE profile with *Sfi*I too. Likewise, single strains that composed groups B, C, and D with *Asc*I differed among them and were assigned to single groups. Clonal relatedness was determined by criteria for interpreting PFGE profiles published by Tenover and colleagues (Tenover et al., 1995). Strains of group A were found to be clonal for both restriction patterns), while strains of groups B and D (according to Tenover’s criteria and percentage of similarity) might be closely related to group prevailing (Tenover et al., 1995).

### RAPD, Rep-PCR, and SAU-PCR typing

**Fig. 3:**
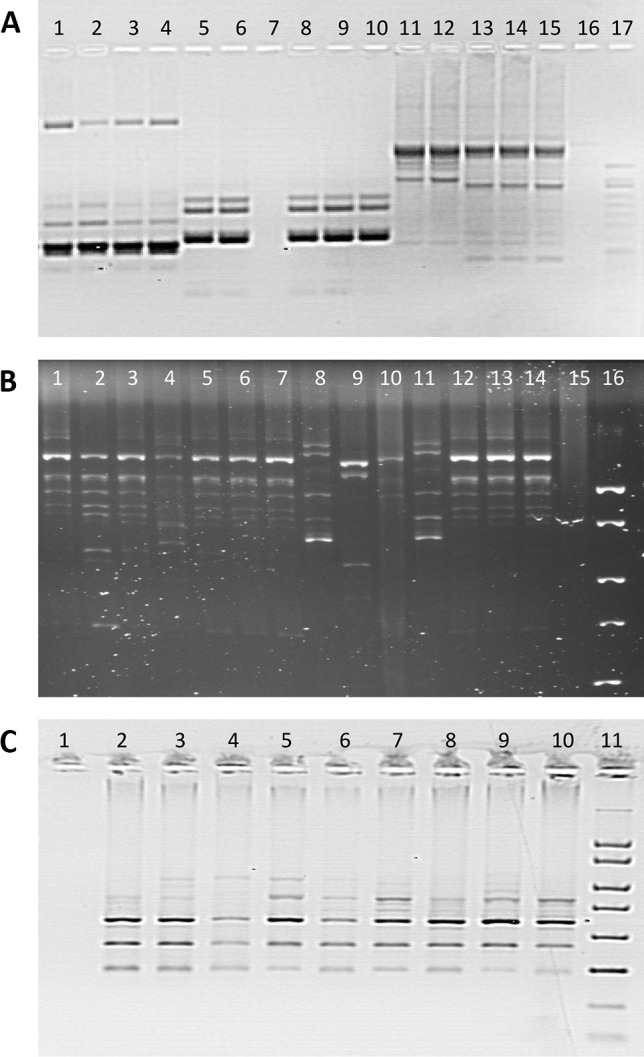
Example of fingerprints obtained using RAPD (panel **A**, lines 1–6 and 8–16, *L. anisa* isolates; line 7, negative control; line 17, molecular marker), Rep-PCR (panel **B**, lines 1–14, *L. anisa* isolates; line 15, negative control; line 16, molecular marker), and SAU-PCR (panel **C**, line 1, negative control; lines 2–10, *L. anisa* isolates; line 11, molecular marker)

Three other genetic fingerprint techniques based on different genetic targets and with different degrees of stringency were applied to confirm the PFGE results and evaluate strain diversity. RAPD, Rep-PCR, and SAU-PCR effectively produced fingerprints for each individual isolate (Fig. 3). Moreover, these techniques showed greater discriminative power than PFGE with restriction enzyme AscI, allowing confirmation that the isolates were individual strains and not clones. However, the use of all three techniques was critical in establishing that the strains were different, but genetically related.

**Fig. 4:**
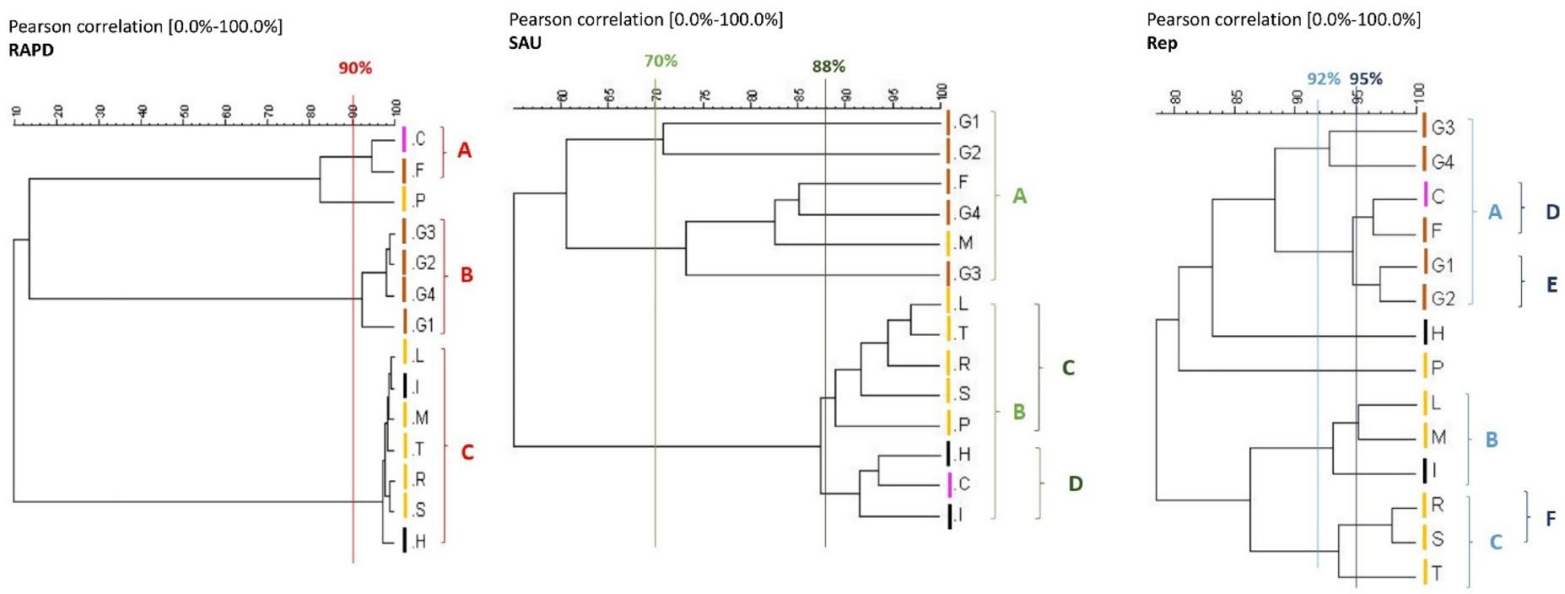
Dendrograms obtained from analysis of the strains’ fingerprints obtained by RAPD, SAU-PCR, and Rep-PCR. The analysis was based on Pearson product-moment correlation coefficients determined using UPGMA clustering algorithms. Clusters (indicated by letters) were reported by the same color of the coefficient of similarity used to define them. Clusters (indicated by letters) were reported by the same color of the coefficient of similarity used to define them. Strains L, M, P, R, S, and T were isolated from pavilion pI  ; C from pavilion pII  ; H and I from pavilion pIII  ; and F, G1, G2, G3, G4 from pavilion pIV

Calculation of similarities in the profiles of bands based on Pearson product-moment correlation coefficients allowed production of dendrograms using UPGMA clustering algorithms (Fig. 4). The RAPD technique allowed discrimination of the strains according to the place of origin. In fact, cutting the cluster at a similarity of 90% resulted in the majority of the strains being grouped into two main clusters, B and C. Cluster B contained all of the strains isolated from pavilion pIV while cluster C included five strains isolated from pavilion pI and two from pavilion pIII. It should be noted that although strain H belonged to group C, it differed greatly from the others. This finding was confirmed by the SAU dendrogram. Using SAU-PCR technique, a higher degree of discrimination among strains emerged. Using a similarity cutoff of 70% resulted in the formation of two large clusters, A and B. Cluster A contained all of the strains isolated from pavilion pIV and a single outlier from pavilion pI (strain M). Although present within cluster A, strain M differed from the others strains within the cluster (82% similarity). This finding agreed with the PFGE results and clearly demonstrated that this strain was one of three that differed from the other strains. Cluster B contained all remaining strains. However, when a cutoff of 88% similarity was used, cluster B broke up into two clusters; namely, cluster C, which contained all strains from pavilion pI, and cluster D, which contained all of the strains isolated from pavilion pIII in one group and a strain isolated from pavilion pII. The Rep-PCR technique confirmed this genetic variability between strains, albeit with different clusters. Specifically, this method denoted a differentiation based on the site of isolation, although there were some strains that were more similar to other isolates at distant points.

## Discussion

The best approach to prevent waterborne diseases is systematic environmental monitoring and accurate identification of strains circulating in water distribution systems coupled with implementation of a risk assessment plan. Over the years, this approach has improved in hospitals, especially for the control and prevention of legionellosis (Mazzotta et al., 2021).

Culture-based investigations still represent the most used method for the isolation of *Legionella* strains, followed by serological agglutination tests with poly/monovalent antisera (Linee guida per la Prevenzione e il Controllo della Legionellosi, 2015; ISO 11731:2017). However, while serological tests can identify *Lpn* and its serogroups, they cannot identify all of the *L-np* species. Particularly problematic characteristics of these tests are that the monovalent antiserum for *L. anisa* is currently not commercially available and anti-*L. bozemanii* antiserum shows cross-reaction between *L. bozemanii* and *L. anisa* (Tateyama, 1992).

Over 13 months, we repeatedly isolated *L-np*, which were initially identified as *L. bozemanii*, by the slide agglutination test. Because these strains had not previously been isolated frequently in our hospital, we confirmed their identification by MALDI-TOF and *mip*-gene sequencing (Dilger et al., 2016; Ratcliff et al., 1998). This approach allowed elucidation of the preliminary misidentification of the strains, which resulted as *L. anisa*. Both species showed blue-white fluorescence when exposed to a wood lamp.

Alternative methods to slide agglutination tests (i.e., molecular methods) are generally only used during epidemiological investigations, not in routine environmental surveillance (Ratcliff et al., 1998).

The misleading identification of *L. bozemani* in place of *L. anisa* demonstrates the importance of correct identification of environmental strains, particularly those belonging to *L-np* species. In addition to ecological relevance, accurate phylogenetic characterization of *Legionella* contamination is essential to assessing its potential impact on the etiology of nosocomial pneumonia. Indeed, the importance of such information is widely recognized to acquire correct and complete epidemiological data and, consequently, design effective strategies to control legionellosis (Rota et al., 2020).

Although the pathogenicity of *L. anisa* is considered low, it has been reported that *Lpn* serogroup (sg) 1 is not the only species or serogroup responsible for clinical cases and other species can cause human pathologies (Cross et al., 2016; Montagna et al., 2007). In several countries, including Italy, *L. anisa* has been often identified as one of the most abundant contaminating species (Federici et al., 2017; Mazzotta et al., 2021). Furthermore, *L. anisa* can mask *Lpn* contamination in the water supply (Orsini et al., 2011; van der Mee-Marquet et al., 2006). In our study, the concentration of *L. anisa* was higher than that of *Lpn* sg 1 when mixed cultures were detected. Our findings also confirmed that higher water temperatures affect the load of *Lpn*, with lower temperatures favoring the presence of *L-np* (e.g., *L. anisa*) (Girolamini et al., 2022). This phenomenon suggests the need for new diagnostic approaches capable of widespread identification even of *L-np*. Currently, such identification can be performed by MALDI-TOF analysis and *mip*-gene sequencing (Dilger et al., 2016; Ratcliff et al., 1998) displaying a high concordance level (Pascale et al., 2020).

Epidemiological investigations uncover different aspects of *Legionella* contamination because the goal of such studies is identification of the source of infection to enable its control (Mazzotta et al., 2021). PFGE is one of the molecular techniques indicated for comparison of the genomic profiles among *Legionella* strains of different origin by the National Guidelines (Linee Guida per la Prevenzione e il Controllo della Legionellosi, 2015). However, our results suggest that the use of other molecular techniques could be a useful tool that more accurately characterizes the identity of circulating *Legionella* strains, according to the results found in other studies (Haroon et al., 2010; Yamamoto et al., 2003; Yan et al., 2008).

The unusual presence of *L. anisa* strains in the water supply of the four investigated pavilions indicated that understanding the genetic variability of the isolated strains was necessary to determine if the hospital was contaminated with one or more different *L. anisa* strains. PFGE, which is known as a laborious and time-consuming technique (Dilger et al., 2016), was initially used. This was followed by RAPD, rep-PCR, and SAU-PCR analysis, which are rapid, simple, reproducible, and inexpensive (Haroon et al., 2010). Although these techniques are not usually applied to *L-np* (Haroon et al., 2010; Corich et al., 2005; Yamamoto et al., 2003), they demonstrated a greater discriminative power than PFGE, allowing us to establish that not all of the isolated strains belonged to the same clone. In addition, genetic variability between the strains denoting a differentiation based on the site of origin was highlighted.

The reason about genetic differences among the strains from different pavilions is still unclear. Diverse eras of pavilion construction, different entry points of the water network, water temperatures, disinfection systems, or pipe materials could be responsible for the genetic variability (Girolamini et al., 2022). The presence of pavilion-specific clusters indicates the rooting of strains that have become characteristic for particular isolation sites. This phenomenon may depend on the natural selection of strains adapted to the specific environmental conditions in which they have been isolated (Akermi et al., 2006).

If extended to the entire hospital water network, our study could allow development of a map of *Legionella* distribution, which would enable investigation of variability in terms of strain diversity between environments. The study of *Legionella* belonging to *L-np* could improve knowledge regarding less-documented species in hospitals and other surveillance protocols (Mazzotta et al., 2021). Moreover, a regional map of *Legionella* will support control and prevention of *Legionella* and it will allow to follow the evolution of the strains over time which is affected by different factors such as mutations, resistance, and pathogenicity (Mazzotta et al., 2021).

Further investigations are still necessary to investigate the role of molecular typing in the environmental surveillance of a water network contaminated by *L-np*.

## Conclusions

Understanding the distribution and interaction between different *Legionella* species is valuable for obtaining and programming the correct strategies to control these bacteria (e.g., temperature values or concentrations of disinfectants), as well as for evaluation of the efficacy of preventive strategies applied. Moreover, accurate information regarding the relationships among isolates over time and during the disinfection treatment is helpful in the comprehension of the dynamics of *Legionella* contamination, in order to promptly take effective corrective measures.

## Data Availability

All data generated or analyzed during this study are included in this published article.

## Declarations

All authors have read, understood, and complied as applicable with the statement on “Ethical responsibilities of Authors” as found in the “Instructions for Authors.”
